# Development and validation of a photogrammetry-based preoperative method (BREAST-E) for quantitative breast morphometric analysis and volume estimation of breast in reconstructive surgery

**DOI:** 10.1371/journal.pone.0353970

**Published:** 2026-07-28

**Authors:** Mee Hoong See, Kwan Hoong Ng, Teng Aik Ong, Lai Kuan Wong, Lee Lee Lai, Sheng Pu Teo, Jing Hui Ng, Jia Min Tneoh, Mahmoud Danaee

**Affiliations:** 1 Breast and Endocrine Surgery Unit, Department of Surgery, Faculty of Medicine, Universiti Malaya, Kuala Lumpur, Malaysia; 2 Surgery Department, Faculty of Medicine, Universiti Malaya, Kuala Lumpur, Malaysia; 3 Department Biomedical Imaging, Faculty of Medicine, Universiti Malaya, Kuala Lumpur, Malaysia; 4 Faculty of Medicine and Health Sciences, UCSI University, Springhill, Negeri Sembilan, Malaysia; 5 Faculty of Computing and Informatics, Multimedia University, Cyberjaya, Selangor, Malaysia; 6 Department of Social and Preventive Medicine, Faculty of Medicine, Universiti Malaya, Kuala Lumpur, Malaysia; University of Perugia: Universita degli Studi di Perugia, ITALY

## Abstract

**Background:**

The demand for reproducible, reliable, and cost-effective tools regarding preoperative breast surgery planning is significant in low- and middle-income countries (LMICs) due to limited advanced imaging access, insufficient standardisation, and high cost. This study developed the BREAST-E technique for accurate and standardised breast volume analysis.

**Methodology:**

A photogrammetry method assessed four ptosis levels: (i) no-ptosis, (ii) mild, [Watterson PA, et al. Plastic and Reconstructive Surgery. 1995;95:1185–94] moderate, and (iv) severe. Key parameters included marker counts (20, 40, and 60), photo angles (10°, 20°, and 30°), and offset adjustments (−0.01, 0, and +0.01). Two-dimensional (2D) images were converted into 3D models via Photomodeler for volume estimation. Clinical validation involved 12 patients (13 breasts) undergoing mastectomy, comparing estimated breast volumes to mastectomy specimens. The primary endpoint was accuracy of volume estimation compared with mastectomy specimen volume, while secondary endpoints included reliability, agreement, and workflow feasibility.

**Results:**

The BREAST-E technique yielded promising accuracy [Pearson correlation coefficient (*R*) = 0.899 and mean absolute error (MAE) = 98.12 mL] and encouraging reliability (ICC = 0.995–0.996) across ptosis levels. Bland-Altman analysis indicated negligible bias, while Cronbach’s alpha (reaching 0.994) demonstrated robust internal consistency. Clinical validation also implied strong performance (*R* = 0.789, MAE = 104.69 mL, and mean error = −27.00 mL). Consequently, no-ptosis presented the highest reliability (ICC = 0.81 and Cronbach’s alpha = 0.92) with minimal bias, whereas moderate and severe ptosis levels demonstrated greater variability.

**Conclusion:**

This study successfully indicated that a reproducible, accessible, and cost-effective method for analysing breast volume was accomplished through the proposed BREAST-E technique for LMICs.

## Introduction

Breast cancer emerged as the most diagnosed cancer worldwide in 2020, accounting for 2.3 million new cases (or 11.7% of all cancer diagnoses). This phenomenon resulted in 685,000 deaths, positioning it as the fifth leading cause of cancer-related mortality. The age-standardised incidence rate was also 47.8 per 100,000 women globally, with elevated rates noted in developed regions. Thus, this data revealed notable regional disparities, emphasising the necessity of addressing inequalities in breast cancer prevention and treatment [1–3].

Preoperative assessment of breast volume and morphology is critical for achieving optimal reconstruction outcomes to enhance aesthetics and patient satisfaction [4–8]. Although conventional techniques are precise, they are often uncomfortable and impractical [9–11]. One prominent example is the water displacement method [12]. Anthropometric techniques are also cost-effective alternatives to achieve these outcomes [13]. Nevertheless, these strategies exhibit inconsistencies attributed to the variability in breast shapes [13–15].

Another common but unreliable approach involves estimating breast volume based on bra cup size, which has demonstrated poor reliability [16,17]. Numerous advanced imaging techniques also provide high precision [18–20]. Nonetheless, these approaches are often prohibitively expensive and unavailable in low-resource environments. Examples of these advanced technologies include magnetic resonance imaging (MRI), computed tomography (CT), and three-dimensional (3D) laser scanning. Mammography and 3D ultrasound may provide practical means to evaluate breast shape, size and volume in a clinical setting, but the data may be insufficient, and the protocols used are still being studied to validate the results [21]. On the other hand, computed tomography (CT) [22], MRI [22]and 3D laser scanning [23] are widely recognised for their accuracy in providing detailed morphometric data, but they come with significant drawbacks, including high cost, reliance on specialised facilities and lack of portability. Hence, the constraints presented by all these methods suggest an urgent need for affordable and portable solutions.

Closed ranged photogrammetry-based methods frequently observe several advantages, such as non-invasiveness, cost-effectiveness, and user-friendliness [22,24,25].These features render photogrammetry-based methods appropriate for various clinical applications, including maxillofacial surgery, cleft lip or palate correction, and neurosurgery. Generally, multiple two-dimensional (2D) photographs taken from different angles are employed in these methods to create accurate 3D models [26]. Notably, photogrammetry offers a cost-effective solution for preoperative planning within breast reconstructive surgery in LMICs [27]

Multiple technologies (stereophotogrammetry [26], laser scanning [26], and structured light scanning) can facilitate detailed imaging [28]. Nevertheless, these limitations imply that standardised imaging processes and high-resolution systems are essential for enhancing consistency, precision, and practical clinical integration for improved surgical outcomes [15]. Therefore, the study evaluated the BREAST-E technique’s performance in surgery, with the primary endpoint being the accuracy of photogrammetry-based breast volume estimation versus intraoperative specimen volume. Secondary endpoints assessed measurement reliability, agreement across repeated analyses, and workflow feasibility for routine preoperative use. These endpoints aimed to determine whether the method provides clinically meaningful and reproducible measurements. The focus of this study is to evaluate the clinical applicability of a practical volumetric assessment tool rather than to present a purely technical optimisation framework.

## Methodology

### Patient recruitment, selection, and clinical assessment

The University of Malaya Medical Centre Research Ethics Committee (UMREC ID: 202032−8339) sanctioned this study. The clinical validation cohort consisted of 12 patients (13 breasts) undergoing mastectomy for breast cancer at a tertiary care centre. Eligible participants were adults aged 18 years or older who provided informed consent and were able to undergo preoperative imaging procedures. Patients with prior breast surgery that significantly altered breast anatomy, inability to maintain the required imaging position, or incomplete clinical data were excluded from the study.

Patient demographic and clinical information, including age, tumour diagnosis, and relevant clinical characteristics, were recorded. The majority of cases comprised histologically confirmed breast carcinoma, such as invasive ductal carcinoma, with staging determined according to standard clinical assessment where available. These data were collected to ensure appropriate clinical context for evaluating the performance of the proposed method.

**Fig 1:**
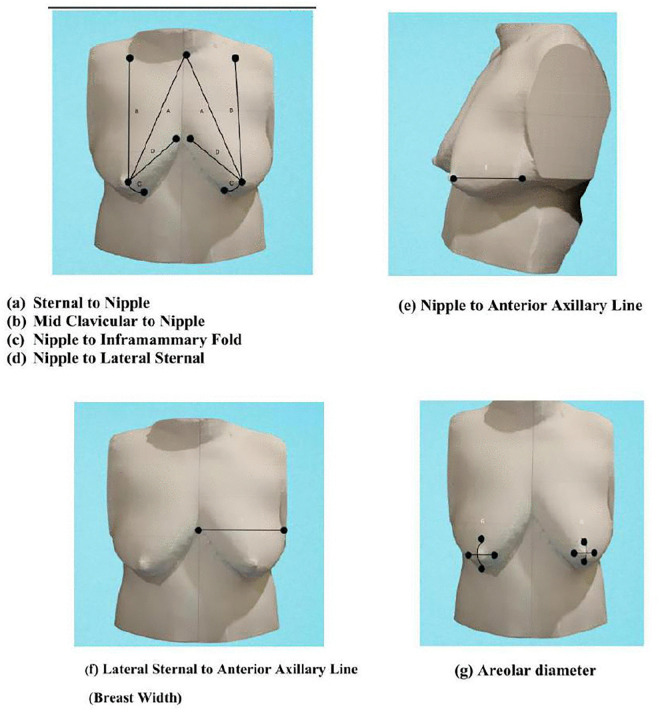
The anthropometric measurements for the breast involving (a) sternal-to-nipple, (b) mid-clavicular-to-nipple, (c) nipple-to-inframammary fold, (d) nipple-to-lateral sternal, (e) nipple-to-anterior axillary line, (f) lateral sternal-to-anterior axillary line, and (g) nipple-areolar complex diameter.

The clinical evaluations also comprised ptosis classification and anthropometric measurements, including sternal notch-to-nipple and nipple-to-inframammary fold distances (see Fig 1). Consequently, the development of accurate 3D breast anatomy models was facilitated by combining these measurements with high-resolution clinical images. Several purposes were then served by this proposed BREAST-E technique, such as enhanced preoperative planning, personalised reconstruction strategies, and optimal aesthetics with functional outcomes specific to the individual characteristics of each patient.

Mastectomy specimen volume was measured immediately after surgery using the water displacement method. This approach was selected to minimise the effects of tissue dehydration and provide an accurate reference for validating breast volume estimations.

### 3D printing of breast model phantom

A 3D printing technology utilising real patient data produced four breast phantom models that reflected varying ptosis levels. The structural accuracy was then facilitated using polylactic acid (PLA) material and a Raised N2 Plus printer (configuration = 0.3 mm layer height and 10% infill). Given that preoperative planning and breast reconstruction simulations relied on lifelike models, skin simulation with improved realism was also established through several post-processing phases. These phases included surface smoothing and beige painting.

### Image acquisition using photogrammetry

**Fig 2:**
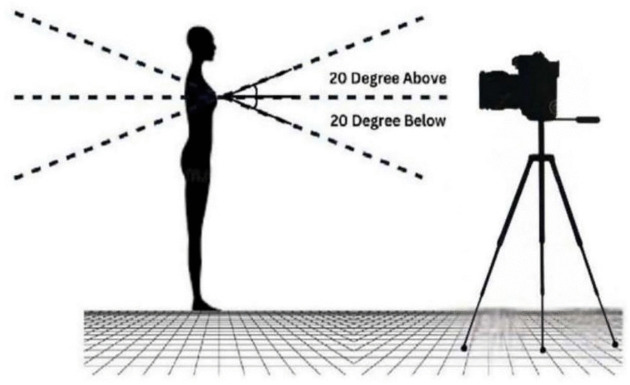
The methodology employed for 2D photogrammetry techniques based on three distinct angles of the photo shooting process (0° level of the nipple, 20° above the nipple, and 20° below the nipple).

A digital single-lens reflex (DSLR) camera mounted on a tripod was utilised in the photogrammetry process to obtain 2D breast surface images from multiple angles and three elevation levels. This image collection process ensured thorough coverage for precise modelling. The three elevation levels are as follows (Fig 2):

i 0° level (straight-on): The camera was positioned at breast height. Images were captured at 10° intervals from 10° to 90° on both sides.
ii 20° elevation (above): The camera was elevated 20° above breast height. Upper breast contour-related images were recorded concerning projection and upper surface details.
iii 20° elevation (below): The camera was positioned 20° below breast height. Inframammary fold and lower breast contour-related images were obtained.

**Fig 3:**
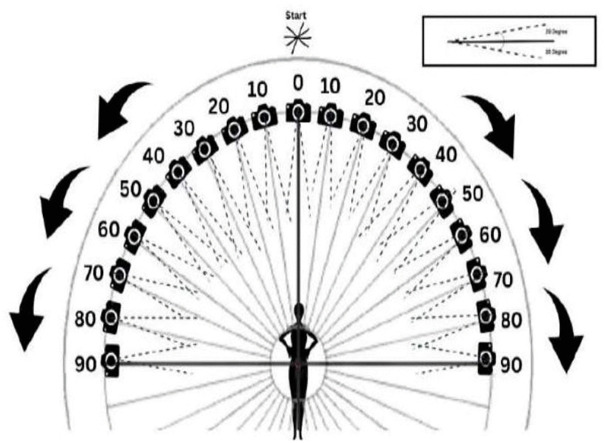
The camera captured images at 10° intervals, encompassing 180° around the patient from both left and right sides for thorough breast assessment.

**Fig 4:**
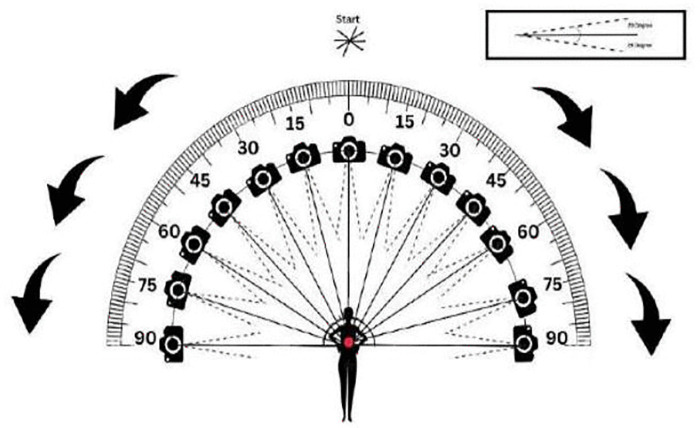
The camera captured images at 15° intervals, encompassing 180° around the patient from both left and right sides for thorough breast assessment.

Each elevation required capturing 57 images (10° intervals) (Fig 3) or 39 images (15° intervals) (Fig 4) to obtain a complete 180° view. Figs 2 to 4 depict the methodology and angles employed. Consequently, this approach facilitated precise modelling of the breast surface.

#### Marker application and anatomical points.

**Fig 5:**
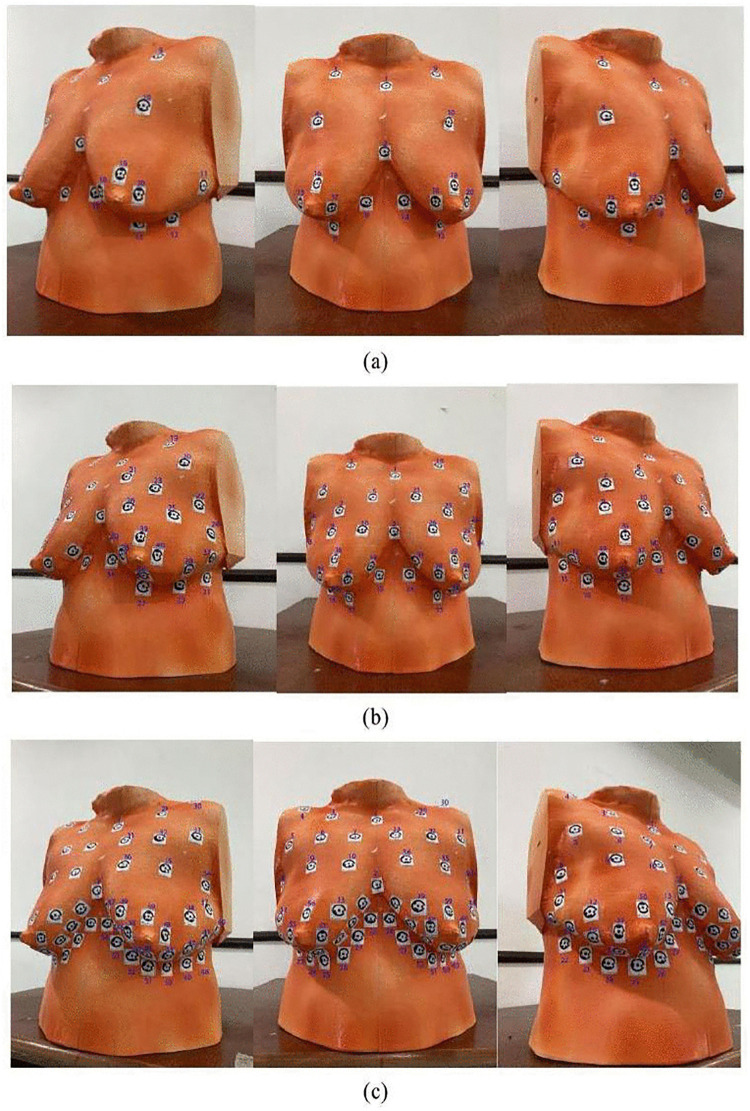
The marker placements on the model involving (a) 20, (b) 40, and (c) 60 markers.

Several adhesive marker counts (20, 40, and 60) from the Photomodeler software were strategically positioned on essential anatomical landmarks and the breast surface area for accurate 3D reconstruction (see Fig 5). These markers served as critical reference points for image alignment. Particularly, key placements comprised the sternal notch (superior sternum border for vertical reference), second intercostal level (vertical and horizontal calibration), nipple-areolar complex (centre of nipple and areola borders for measurement accuracy), and inframammary fold (natural fold defining the lower boundary of the breast). This strategic marker application enabled precise alignment and dependable reconstruction throughout imaging.

#### 3D model reconstruction and digital sculpting.

Images were processed with the Photomodeler software, which integrated 2D images to create a detailed 3D breast surface model. The markers functioned as reference points, allowing the sophisticated software algorithms to accurately align images based on their positions and anthropometric data. Subsequently, digital sculpting tools were applied to enhance the model, ensuring an accurate representation of breast tissue and different ptosis levels.

#### Post-processing and volume estimation using photomodeler.

The Photomodeler software was employed to analyse the 3D-printed phantom models (four ptosis levels) regarding breast volume and shape. A calibration process was also performed to align the models with actual anatomy. In contrast, surface contours were improved by post-processing. The software then delivered precise volumetric measurements critical for preoperative planning in breast reconstruction.

#### Clinical validation.

Three patients from each ptosis category were used for clinical validation. A comparative analysis was conducted between the clinical measurements and several modelling outcomes (image acquisition, 3D reconstruction, and printing). These measurements included the sternal notch-to-nipple distance, breast width, nipple-to-inframammary fold distance, breast height, and mastectomy-derived volumes. Therefore, the proposed BREAST-E technique was enhanced by this analysis while validating its clinical applicability.

### Data analysis

Based on statistical analysis, a comparative study was performed between the breast volume estimations (accuracy, reliability, and consistency) and actual mastectomy specimen volumes. This assessment was executed utilising Statistical Package for the Social Sciences (SPSS) version 26 (IBM Corp, Armonk, NY, USA) and MedCalc 23.0.6 (MedCalc Software Ltd., Ostend, Belgium). Meanwhile, the linear relationship between estimated and actual volumes for assessing the alignment with high values was determined through the correlation coefficients. The employed correlation coefficients are as follows:

i Accuracy metrics: These metrics involved the mean absolute error (MAE) and root mean squared error (RMSE) to assess the degree to which estimates aligned with actual measurements. A higher accuracy was indicated if lower values were presented.
ii Bland-Altman analysis: Precision insights were demonstrated by this analysis by evaluating agreement and identifying any systematic bias.
iii Intraclass correlation coefficient (ICC): The reliability across four ptosis levels and 27 photogrammetry subgroups based on the various combinations of markers, photo angles, and offsets in the Photomodeler software was examined through this metric.
iv Internal consistency: The robustness of internal consistency across subgroups was investigated utilising Cronbach’s alpha.

This study classified ptosis based on four levels: (i) Level 0 (no-ptosis), (ii) Level 1 (mild), (iii) Level 2 (moderate), and (iv) Level 3 (severe). The classification process followed the position of the nipple concerning the inframammary fold. Subgroups were also examined to determine optimal configurations for photogrammetry-based 3D modelling concerning precision, reproducibility, and processing efficiency. Furthermore, a *p*-value less than 0.05 and 95% confidence intervals less than 1.0 indicated statistical significance.

The primary clinical endpoint of this study was the accuracy of photogrammetry-derived breast volume measurements relative to mastectomy specimen volumes. Secondary endpoints included assessment of reliability, agreement, and feasibility of the imaging workflow. Accuracy was evaluated using error metrics and correlation analyses, reliability was assessed using intraclass correlation coefficients, and agreement between methods was examined using Bland–Altman analysis. These analyses were performed to determine the robustness and clinical applicability of the proposed BREAST-E technique.

The exploration of multiple parameter combinations, including marker counts, imaging angles, and software offsets, was conducted to identify the optimal configuration that balances accuracy, reliability, and practicality (Appendix). This systematic evaluation was intended for methodological optimisation rather than routine clinical use. In clinical practice, only the recommended protocol derived from this analysis is required, thereby minimising procedural complexity and reducing the burden on clinical staff while maintaining measurement performance.

## Results

The performance of the BREAST-E technique is presented using key metrics reflecting accuracy, reliability, and agreement to facilitate clinical interpretation. Detailed exploratory analyses are provided in the Supplementary Material.

### Development phase: 3D phantom

This study applied a 3D phantom model that included four ptosis levels (no-ptosis, mild, moderate, severe) and 27 subgroups. The subgroups integrated variables of marker count (20, 40, and 60), photo angles (10°, 20°, and 30°), and offset adjustments (−0.01, 0, and +0.01) for estimating breast utilising the Photomodeler software. All combinations for breast volume estimation were incorporated in each case. The estimated breast volumes were then analysed and compared to intraoperative mastectomy breast volumes, which were categorised by subgroup (see Appendix I).

**Fig 6:**
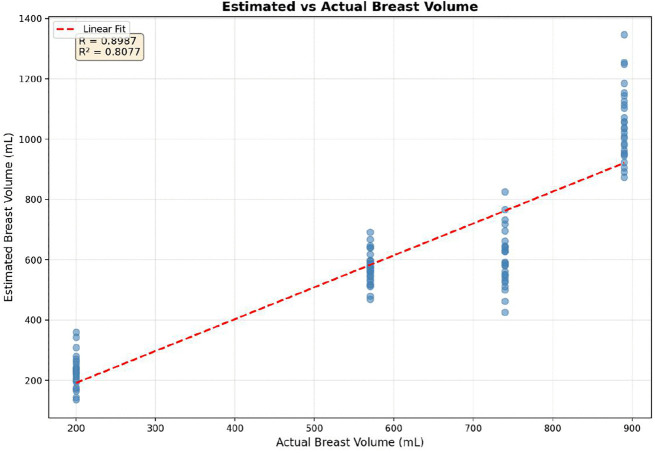
Scatter plot showing the correlation between estimated and actual breast volumes for the clinical validation cohort (3D Phantom cohort).

The 3D phantom study demonstrated the accuracy of the proposed BREAST-E technique in photogrammetry-based breast volume estimation. This metric was essential for preoperative planning in reconstructive surgery. A strong linear relationship was then exhibited by the Pearson correlation coefficient (*R* = 0.899), with 80.8% of the variance in actual volume accounted for by estimated values (see Fig 6). The precision of the proposed BREAST-E technique also revealed an MAE of 98.12 and a mean error (ME) of 14.44, which were accompanied by standard deviations (SDs) of 92.04 and 134.09, respectively. Thus, the reliability and precision of the proposed BREAST-E technique were verified based on the outcomes, indicating accurate breast volume estimation in clinical applications.

The examination of breast volume estimation at varying ptosis levels demonstrated significant differences in consistency and accuracy as follows (see Table 1):

**Table 1:** Summary of the MAE, SD, and MPE results categorised by ptosis levels based on the phantom model.

Ptosis Level	Mean Absolute Error (mL)	Standard Deviation (mL)	Mean Percentage Error	Range (mL)	Outcome
Level 0 (No Ptosis)	48.17	45.11	48.17%	−56.22 to 159.02	Best overall accuracy, moderate variability, slight positive bias, and most consistent measurements.
Level 1 (Mild)	60.72	43.76	60.72%	−101.33 to 121.23	Increased error compared to Level 0, similar standard deviation, near-neutral bias, and wider range of errors.
Level 2 (Moderate)	121.72	67.48	121.72%	−209.53 to 27.25	Significant increase in error, consistent underestimation, higher variability, and large negative bias.
Level 3 (Severe)	228.77	122.26	228.77%	60.04 to 455.86	Highest error rates, consistent overestimation, largest variability, and substantial positive bias.

i Level 0 (no ptosis): This level revealed the highest overall accuracy, with an MAE of 48.17 mL and an SD of 45.11 mL. The measurements exhibited moderate variability, slight positive bias, and the highest consistency across all ptosis levels. Therefore, good reliability was indicated for clinical applications.
ii Level 1 (mild): The errors increased relative to Level 0, with an MAE of 60.72 mL and a SD of 43.76 mL. Despite that the bias remained neutral, the error range expanded. This outcome signified a minor decrease in consistency.
iii Level 2 (moderate): The errors were notably elevated, yielding an MAE of 121.72 mL and an SD of 67.48 mL. Additionally, the persistent underestimation and increased variability underscored challenges within this category, resulting in diminished consistency of results.
iv Level 3 (severe): This level displayed the highest error rates, with an MAE of 228.77 mL and an SD of 122.26 mL. Nonetheless, the limitations of the method in this category were evidenced by consistent overestimation, significant variability, and considerable positive bias. This finding could impact consistency.

The assessment of breast volume estimations across varying ptosis levels was performed by analysing mean differences, limits of agreement, ICC, and Cronbach’s alpha (see Table 2). Consequently, Level 0 (no-ptosis) depicted the highest Cronbach’s Alpha (0.914) and excellent reliability (ICC = 0.995) with minimal variability. Level 1 (mild) presented the highest ICC (0.997) and slightly increased variability while maintaining consistency. Level 2 (moderate) also computed the lowest mean difference. Nonetheless, increased variability and reliability (ICC = 0.996) were observed. Level 3 (severe) finally reported the most significant mean difference (14.754 mL) and the widest limits of agreement. Conversely, high reliability (ICC = 0.996) was preserved. Even though the escalation in ptosis severity was denoted, this outcome suggested a high robustness.

**Table 2:** Summary of Bland-Altman agreement, ICC, and Cronbach’s alpha results categorised by ptosis levels based on the phantom model.

Ptosis Level	Mean Difference (mL)	Limits of Agreement (mL)	ICC	Cronbach’s Alpha	Summary
No Ptosis	0.223	−18.375 to 18.821	0.995	0.914	Highest mean Cronbach’s Alpha with relatively low variability, Excellent reliability with very small variation.
Mild Ptosis	7.137	−27.346 to 41.620	0.997	0.873	Slightly lower mean but still within a high range, Highest ICC value among all groups; most consistent measurements.
Moderate Ptosis	6.541	−47.491 to 60.573	0.996	0.851	Lowest mean with slightly higher variability, very high reliability maintained.
Severe Ptosis	14.754	−61.284 to 90.791	0.996	0.889	Mean of 0.889 with the least variability among the groups, consistently high reliability despite severity.

**Fig 7:**
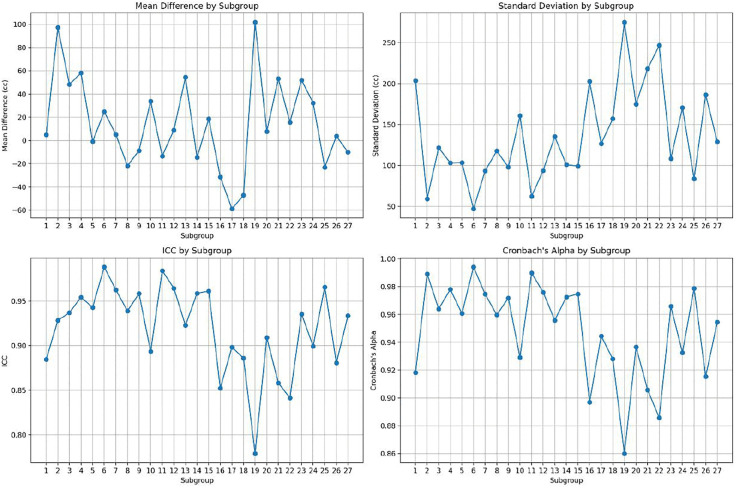
The graphs involving the (a) mean difference, (b) SD, (c) ICC, and (d) Cronbach’s alpha categorised by subgroup based on the phantom model.

Subgroup 6 (20 markers, 20°, and 0.01 offset) exhibited superior best performance compared to all other groups, achieving the highest ICC (0.988) and Cronbach’s alpha (0.994). This observation indicated outstanding reliability and internal consistency. The analysis also recorded a low mean difference (24.721 cc) and the smallest SD (47.053 cc), demonstrating high accuracy and minimal measurement variability. Furthermore, the overall score of 0.941 was the highest, implying superior balance across all evaluation criteria. Consequently, these results signified that Subgroup 6 was the most reliable and precise group for 3D phantom analysis. This outcome established the group as the optimal choice for accurate and consistent measurement outcomes (see Fig 7).

### Clinical validation phase

#### Clinical cohort.

Thirteen specimens were analyzed, and all patients had invasive breast carcinoma undergoing mastectomy. Mean age was 56.62 years (41–73) and mean BMI 26.61 (19.17–36.57), with mean height 158.38 cm (149–169) and weight 66.69 kg (46–89). Mean menarche age was 12.77 years (11–15). Histories of breastfeeding, smoking, alcohol intake, and hormonal use were recorded. Cancer staging showed that most patients were Stage II (n = 8), followed by Stage I (n = 2), Stage III (n = 1), and Stage IV (n = 1). Right mastectomy specimens (n = 9) had a mean weight of 463.78 g (184–812) and mean volume of 544.44 ml (200–900), while left specimens (n = 6) had a mean weight of 477.83 g (168–605) and mean volume of 568.33 ml (210–900).

The clinical validation included three patients for each ptosis level, ensuring comprehensive and reproducible workflows. The cohort reflects a pilot clinical sample intended to evaluate feasibility and preliminary performance. Images were then obtained at intervals of 10°, 20°, and 30° across three sets: (i) at the same level, (ii) 20° lower, and (iii) 20° higher. Marker sets consisting of 20, 40, and 60 markers were also utilised to improve the accuracy of 3D models. All datasets were processed using the Photomodeler software with point cloud technology. Three offset settings were implemented to enhance volume estimates, facilitating a comprehensive comparison across different ptosis levels.

**Fig 8:**
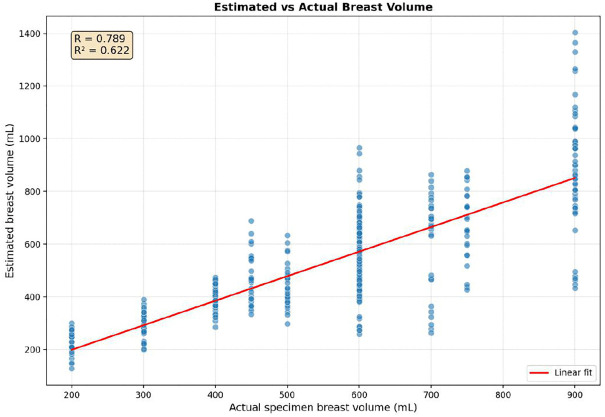
Scatter plot showing the correlation between estimated and actual breast volumes for the clinical validation cohort (clinical validation testing cohort).

The validation revealed a significant correlation between estimated and actual breast volumes, with an *R* of 0.789 and *R*² of 0.622 (see Fig 8). This finding suggested that actual volumes could account for 62.2% of variability in estimated volumes. The regression slope (0.932) and intercept (12.380 mL) were also strongly aligned. Likewise, the mean percentage error (17.83%), RMSE (141.22 mL), MAE (104.69 mL), and mean error (−27.00 mL) highlighted moderate deviations. These outcomes confirmed the high suitability for clinical applications. Thus, the reliability of the workflow as a preoperative planning tool was confirmed based on the obtained data to ensure consistency and applicability across different ptosis levels. The significant correlation and acceptable error metrics also illustrated the potential of the proposed BREAST-E technique to improve clinical breast reconstruction planning.

The results indicated differing degrees of reliability and consistency among ptosis levels. Level 0 (no-ptosis) exhibited the highest reliability (ICC = 0.81) and consistency (Cronbach’s alpha = 0.92), with a mean difference of −13.27 mL. These outcomes suggested a slight underestimation while establishing the benchmark for clinical application. Level 1 (mild) portrayed moderate reliability (ICC = 0.48) and good consistency (Cronbach’s alpha = 0.78). Nonetheless, a mean difference of +43.04 mL was observed, indicating systematic overestimation and necessitating careful interpretation. Level 2 (moderate) had the lowest reliability (ICC = 0.34) and consistency (Cronbach’s alpha = 0.65), with the greatest overestimation (+48.05 mL) and significant bias. These outcomes rendered the level the most challenging category. Lastly, Level 3 (severe) demonstrated moderate reliability (ICC = 0.51) and good consistency (Cronbach’s alpha = 0.78), with a mean difference of +16.16 mL. Therefore, moderate overestimation was indicated. Although an improvement in moderate ptosis was observed, further refinement was pivotal to enhance accuracy in this category. Overall, the results signified the strengths and areas for enhancement within the proposed BREAST-E technique (see Table 3).

**Table 3:** Summary of Bland-Altman agreement, ICC, and Cronbach’s alpha results categorised by ptosis levels based on the clinical validation cohort.

Category	ICC	Cronbach’s Alpha	Bland-Altman Agreement	Outcome
No Ptosis	0.81 (Highest)	0.92 (Highest)	Mean Difference: −13.27 mL (slight underestimation)	Best reliability and consistency; gold standard for clinical use.
Mild Ptosis	0.48 (Moderate)	0.78 (Good)	Mean Difference: + 43.04 mL (overestimation)	Moderate reliability; systematic overestimation; requires careful assessment.
Moderate Ptosis	0.34 (Lowest)	0.65 (Lowest)	Mean Difference: + 48.05 mL (largest overestimation)	Poor reliability; highest bias; most challenging for accurate measurement.
Severe Ptosis	0.51 (Moderate)	0.78 (Good)	Mean Difference: + 16.16 mL (moderate overestimation)	Improved reliability over moderate ptosis; moderate bias; potential for refinement.

**Fig 9:**
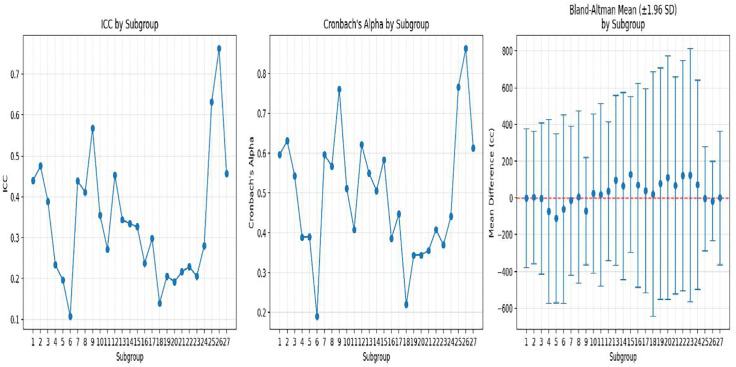
The graphs involving the (a) ICC, (b) Cronbach’s alpha, and (c) Blind Altman mean categorised by subgroup based on the clinical validation cohort.

Subgroup 25 (60 markers, 30°, and offset 0) demonstrated superior performance in the clinical validation cohort, providing an optimal balance of reliability and accuracy. This subgroup attained an ICC of 0.954, indicating excellent measurement consistency. In contrast, the Cronbach’s alpha of 0.971 signified strong internal consistency. The Bland-Altman score of 0.600 also signified good agreement with minimal bias, while the absolute mean error of 2.661 indicated precise estimates (see Fig 9).

Based on the comparative analysis of parameter combinations, a simplified protocol was identified as the recommended configuration for clinical implementation. This protocol demonstrated a favourable balance between accuracy, reliability, and operational efficiency, indicating that high-quality breast volume estimation can be achieved without requiring extensive parameter adjustments. The findings suggest that the streamlined workflow is suitable for routine preoperative assessment.

The BREAST-E technique demonstrated good accuracy, with a strong correlation between estimated and reference volumes and acceptable mean absolute error across ptosis levels. Reliability analysis showed high consistency of measurements, while Bland–Altman analysis indicated minimal systematic bias with clinically acceptable limits of agreement. These findings support the robustness of the method for preoperative assessment.

## Discussion

Historically, an accessible method for clinical volume estimation was achieved through conventional photogrammetry techniques in topography and architecture within LMICs. Nevertheless, an economical and dependable technique was effectively demonstrated in this study for estimating preoperative breast volume and ptosis in mastectomy patients who were receiving immediate reconstruction. This approach facilitated precise 3D reconstructions from 2D images while eliminating direct patient contact. Consequently, discomfort and potential complications associated with conventional techniques could be minimised. The proposed BREAST-E technique also denoted enhanced preoperative breast morphology and volume assessments as an effective tool. This technique successfully facilitated optimal aesthetic results while improving patient satisfaction in breast reconstruction

The structured photogrammetry framework was analysed in this study based on three primary variables: (i) marker count (20, 40, and 60), (ii) photo capture angles (10°, 20°, and 30°), and (iii) software offsets (−0.01, 0, + 0.01). A systematic assessment of accuracy, reliability, and consistency in breast volume estimation was successfully developed through these combinations by transforming 2D images into 3D models. This outcome was delivered utilising the Photomodeler software. Hence, a reliable and economical basis for breast volume analysis and preoperative planning in breast reconstruction was accomplished by improving the precision and adaptability of current photogrammetry techniques.

### Accuracy

The proposed BREAST-E technique exhibited significantly high accuracy in controlled 3D phantom models. This conclusion was evidenced by an *R* of 0.899, *R*² of 80.8%, and MAE of 98.12 mL. The accuracy also recorded a slight reduction (*R* = 0.789, *R*² = 62.2%, and MAE = 104.69 mL) attributable to variability in breast morphology (ptosis and asymmetry). Generally, the MRI is the gold standard for volumetric analysis due to its superior soft-tissue resolution. Nonetheless, this method has remained costly and time-consuming [29,30]. The comparable precision offered by CT volumetrics also reveals several issues [31,32]. One notable example is ionising radiation, which limits their routine application [33]. Therefore, the proposed BREAST-E technique in this study provided a balance of accuracy, cost-effectiveness, and accessibility, rendering it a viable option for breast reconstruction in resource-constrained environments.

### Reliability

The controlled imaging conditions of photogrammetry techniques demonstrate their significant reliability in the phantom model. Even though the Cronbach’s alpha (0.8749) obtained in this study indicated strong internal consistency, reliability decreased in the actual patient cohort. This outcome was attributed to low ICC (−0.0568) caused by anatomical variability and imaging inconsistencies. Typically, high reliability is observed in MRI and CT scans utilising standardised protocols and accurate internal imaging [32,34]. High reproducibility is also denoted in 3D laser scanners provided via surface mapping [35,36]. Conversely, this tool usually encounters limitations related to cost and equipment [37]. Thus, the proposed BREAST-E technique was presented as a suitable alternative to these tools, implying that workflow refinements are necessary to improve reliability in clinical practice.

### Consistency

The proposed BREAST-E technique exhibited minimal variability in controlled conditions based on the significant consistency in 3D phantom models (SD of the MAE = 92.04 mL). Nonetheless, patient cohorts’ variability increased (SD = 99.57 mL) due to numerous anatomical factors (ptosis, asymmetry, and volume differences). Standardised imaging protocols that reduce error and variability lead to improved consistency in MRI and CT volumetrics showed better consistency [21,38]. On the contrary, inadequate consistency is demonstrated in anthropometric methods that depend on manual measurements for ptotic and asymmetrical breasts. One substantial example is surface metrics frequently do not accurately reflect true volume [34]. This observation indicates that refinements are necessary to address patient-specific variability.

### Agreement

The agreement between estimated and actual volumes was evaluated through Bland-Altman analysis, in which the proposed BREAST-E technique in the 3D phantom model exhibited narrow limits of agreement and minimal systematic bias. Nevertheless, these limits expanded in actual patient cohorts (−251.00 mL to 305.00 mL) with a mean difference of −27.00 mL, suggesting occasional underestimation. Several factors could cause this variability, including breast position, marker placement, and imaging angles. Meanwhile, 3D laser scanners yield superior agreement attributed to their precise surface contour mapping [37,39]. Anthropometric methods also depict limited agreement for irregular or ptotic breasts [34]. Hence, improving photogrammetry workflows can minimise biases and increase accuracy in clinical applications.

### Clinical feasibility and comparison with existing techniques

The proposed BREAST-E workflow may support surgical planning by providing a low-cost, accessible approach for breast volumetric assessment, particularly in settings where advanced imaging modalities are not readily available. By enabling objective preoperative evaluation, the technique has the potential to assist clinicians in decision-making and improve planning for reconstructive procedures. Practical implementation in a clinical environment was assessed with respect to workflow duration and ease of use: marker placement typically requires 5–10 minutes, image acquisition 5 minutes, and computational processing and 3D reconstruction 10–20 minutes, depending on hardware and operator experience. Once familiarity with the protocol is achieved, the workflow can be performed efficiently by trained personnel, and a simplified configuration supports reproducibility across operators.

The distinction between phantom validation and clinical validation is important when interpreting the performance of the BREAST-E workflow. Under controlled phantom conditions, the technique demonstrated excellent reproducibility and agreement, reflecting the technical capability of the photogrammetry system when imaging variables, surface geometry, and marker positioning were standardised. In contrast, the clinical cohort introduced substantially greater variability due to real-world anatomical complexity, including breast asymmetry, soft tissue deformation, ptosis severity, patient positioning, and inconsistencies in marker placement and image acquisition. This effect was particularly evident in moderate and severe ptosis categories, where wider Bland–Altman limits of agreement and lower ICC values suggested reduced measurement stability and greater susceptibility to surface contour distortion. These findings indicate that while BREAST-E demonstrates promising feasibility as an accessible adjunctive preoperative assessment tool, its clinical robustness remains dependent on anatomical conditions and workflow optimisation.

MRI- and CT-based breast volumetric techniques remain the reference standards due to their excellent soft-tissue delineation, high reproducibility, and reported volumetric errors frequently below 5–10%; however, their dependence on specialised infrastructure, longer acquisition times, and substantial operational costs limits routine applicability in many clinical settings, particularly within LMICs [22,29–32]. Although commercial 3D surface imaging systems demonstrate excellent reproducibility with ICC values commonly exceeding 0.90 and provide faster acquisition workflows, their reliance on proprietary hardware and software may still restrict accessibility and scalability in resource-constrained environments [23,35–37]. In contrast, anthropometric methods offer affordability and simplicity but remain highly operator-dependent and are less reliable in ptotic, asymmetrical, or larger breasts where surface landmarks may not accurately reflect true tissue volume [13,15,21]. Within this context, BREAST-E does not aim to replace advanced volumetric imaging modalities but instead provides a pragmatic compromise between accuracy, cost, portability, and clinical feasibility. The observed reduction in reliability in moderate and severe ptosis categories further highlights that anatomical complexity remains a significant challenge for photogrammetry-based approaches and underscores the need for continued workflow refinement and larger-scale clinical validation before broader clinical adoption can be recommended.

### Limitations

The BREAST-E workflow was developed and validated primarily for patients with intact breasts undergoing mastectomy and immediate reconstruction. Its applicability to breast-conserving surgery remains limited. In procedures such as lumpectomy, where only partial breast tissue is removed, future adaptations may require regional surface segmentation, pre- and post-operative model registration, and virtual resection simulation to quantify localized volume changes. Integration with tumour localisation imaging could further refine surgical planning in such scenarios. Another limitation is the relatively small clinical validation cohort, particularly for patients with moderate to severe ptosis. Although this study serves as a proof-of-concept following extensive phantom validation, larger multi-centre studies are needed to improve statistical power and confirm generalisability across diverse patient populations. Additionally, the use of categorical ptosis grading (levels 0–3) provides a clinically practical framework but may not fully capture subtle inter-breast asymmetry or nuanced contour variations that could influence reconstructive decision-making. Future work may incorporate continuous morphometric descriptors, curvature mapping, or surface deviation analyses to better characterise three-dimensional breast morphology. Finally, while the workflow is feasible in a clinical setting—requiring approximately 2–5 minutes for marker placement, 8–12 minutes for image acquisition, and 20–30 minutes for 3D reconstruction and post-processing—occasional variability highlights the need for continued refinement and operator training. Consequently, we interpret the present findings as encouraging preliminary clinical validation rather than conclusive evidence of equivalence to established volumetric imaging modalities. We believe that further large-scale, multicentre studies involving more diverse patient populations will be important to further evaluate the robustness, reproducibility, and broader clinical applicability of the BREAST-E workflow.

### Future directions

Future developments could address the current limitations and broaden the clinical applicability of BREAST-E. Continuous morphometric measures and quantitative surface deviation analyses may provide more precise assessment of breast shape and asymmetry. Adaptations for breast-conserving surgery could include regional surface segmentation, pre- and post-operative registration, virtual resection simulation, and integration with tumour localisation imaging. Emerging technologies such as smartphone-based photogrammetry, artificial intelligence for automated landmark detection, and cloud-based processing platforms may further improve accessibility, reduce operator dependency, and accelerate reconstruction workflows, particularly in resource-limited settings. Larger multicentre studies with diverse patient populations will be important to confirm robustness, reproducibility, and generalisability. Collectively, these efforts can support workflow refinement, protocol optimisation, and broader clinical translation of the BREAST-E technique.

## Conclusions

This study successfully demonstrated the potential of the proposed BREAST-E technique for preoperative breast volume estimation. Enhanced precision and accessibility in surgical planning and breast cancer care in resource-limited settings could be attained by further refinement and user-friendly advancements, including the integration of AI for automated landmark detection and streamlined workflows. Future refinements, including automated landmark detection and streamlined image processing, may further reduce workflow time and enhance usability

## Supporting information

S1 Appendix Table showed ccombinations of photogrammetry parameters used in the experimental design, including marker number, photo-taking angle (°), and positional offset. (DOCX)
