# Supplementary material for: Development and validation of a photogrammetry-based preoperative method (BREAST-E) for quantitative breast morphometric analysis and volume estimation of breast in reconstructive surgery
Source: PLoS One. 2026 Jul 28;21(7):e0353970. doi: 10.1371/journal.pone.0353970 (PMC13411864; doi:10.1371/journal.pone.0353970)
Supplement: S1 Appendix — (DOCX) [file pone.0353970.s001.docx]

Appendix 1: Table show combinations of photogrammetry parameters used in the experimental design, including marker number, photo-taking angle (°), and positional offset

| Subgroup | Marker | Degree of Photo Taking (°) | Offset |
| --- | --- | --- | --- |
| 1 | 20 | 10 | 0 |
| 2 | 20 | 10 | -0.01 |
| 3 | 20 | 10 | 0.01 |
| 4 | 20 | 20 | 0 |
| 5 | 20 | 20 | -0.01 |
| 6 | 20 | 20 | 0.01 |
| 7 | 20 | 30 | 0 |
| 8 | 20 | 30 | -0.01 |
| 9 | 20 | 30 | 0.01 |
| 10 | 40 | 10 | 0 |
| 11 | 40 | 10 | -0.01 |
| 12 | 40 | 10 | 0.01 |
| 13 | 40 | 20 | 0 |
| 14 | 40 | 20 | -0.01 |
| 15 | 40 | 20 | 0.01 |
| 16 | 40 | 30 | 0 |
| 17 | 40 | 30 | -0.01 |
| 18 | 40 | 30 | 0.01 |
| 19 | 60 | 10 | 0 |
| 20 | 60 | 10 | -0.01 |
| 21 | 60 | 10 | 0.01 |
| 22 | 60 | 20 | 0 |
| 23 | 60 | 20 | -0.01 |
| 24 | 60 | 20 | 0.01 |
| 25 | 60 | 30 | 0 |
| 26 | 60 | 30 | -0.01 |
| 27 | 60 | 30 | .01 |
